# Spatio-Temporal Variation and Its Driving Forces of Soil Organic Carbon along an Urban–Rural Gradient: A Case Study of Beijing

**DOI:** 10.3390/ijerph192215201

**Published:** 2022-11-17

**Authors:** Bingrui Liu, Jiacheng Qian, Ran Zhao, Qijun Yang, Kening Wu, Huafu Zhao, Zhe Feng, Jianhui Dong

**Affiliations:** 1 School of Land Science and Technology, China University of Geosciences, Beijing 100083, China; 2 College of Desert Control Science and Engineering, Inner Mongolia Agricultural University, Hohhot 010018, China; 3 Department of Soil System Science, Helmholtz Centre for Environmental Research—UFZ, 06120 Halle (Saale), Germany; 4 Key Laboratory of Land Consolidation, Ministry of Natural Resources, Beijing 100035, China

**Keywords:** soil organic carbon, land use, climate change, GTWR model, urban–rural gradient, Beijing city

## Abstract

Rapid urbanization has reshaped land cover and the ecological environment, potentially improving or deteriorating soil organic carbon (SOC). However, the response of SOC to urbanization has not yet been fully exploited. Herein, by using the land-use transfer matrix, the Sen & Mann–Kendall tests, the Hurst index, and a geographical and temporal weighted regression (GTWR) model, as well as an urban–rural gradient perspective, we assessed the dynamic response of SOC to Beijing’s urbanization from 2001 to2015 and identified the main drivers. The results found that SOC stock decreased by 7651.50 t C during the study period. SOC density varied significantly along an urban–rural gradient, with high value areas mainly being located in remote mountainous rural areas and low value areas mainly being located in urban areas on the plains. There was an uneven variation in SOC density across the urban–rural gradient, with suburban areas (25–40 km away from urban cores) losing the most SOC density while urban areas and rural areas remained relatively unchanged. GTWR model revealed the spatio-temporal non-flat stability of various driving forces. Precipitation, the proportion of forest, the proportion of grassland, the population, distance to the urban center, the slope, and the silt content are the main factors related to SOC stock change. As a result, we suggest policy makers reconceptualize the uneven variation in the SOC between urban and rural areas, emphasize suburban areas as a target for controlling SOC loss, and take into consideration the spatial and temporal heterogeneity of the factors influencing SOC stock when evaluating policies.

## 1. Introduction

Soil is an extremely valuable natural resource that contributes significantly to maintaining the biogeochemical cycle and the global carbon cycle [1,2]. Soil carbon comprises both soil organic carbon (SOC) and soil inorganic carbon (SIC), making it the second largest carbon pool on Earth after the oceans [3]. About ~1550 Pg C of SOC is stored just 1 m below the surface, almost two to three times the amount of carbon stored in living terrestrial vegetation or in the atmosphere [4]. There is no doubt that any small change in the SOC stock can cause huge environmental effects [5]. A recent study reported that about 79% of the world’s countries or regions experienced significant declines in SOC in the first 15 years of the 21st century, with a total loss of ~3.1 Pg C, or about 8% of the total global SOC loss that has occurred since the Holocene era [6]. This means that a large amount of the carbon that has been sequestered for a long time in geological eras has been released into the atmosphere in a very short period of time, exacerbating the global warming trend and putting pressure on human societies and natural ecosystems. Furthermore, as a derived indicator of soil organic matter, SOC is directly and indirectly linked to improvements in human well-being and the UN Sustainable Development Goals (e.g., SDGs 2.3, 2.4, 15.3, 3.9, 6.4, 6.5, 11.3, and 13.2) [3,6,7]. It is the core indicator of soil health and productivity, and helps to assess regional food security and land degradation neutrality [8]. In addition, SOC supports a range of key ecosystem services, such as the food and fiber supply, providing nutrients, absorbing pollution, conserving soil and water, etc. [9,10,11]. Monitoring and assessing the development of the SOC stock can therefore provide stakeholders with key information to improve our understanding and the management of SOC, which is essential for promoting global and regional sustainable development.

Recently, changes in the SOC stock have received growing attention from the scientific community [2,6], and the mapping of SOC is a prerequisite for conducting SOC stock studies [12]. The development of models and algorithms has advanced digital mapping techniques, with a gradual shift from traditional spatial interpolation to soil spectral data inversion and prediction through machine learning frameworks in combination with environmental covariates [7,11,12,13]. Based on these methods and models, many studies have been carried out to assess the spatial distribution patterns of the SOC stock at different scales [14,15,16], although most of them are limited to specific historical periods. For example, Yu et al. [14] used data from the second Chinese soil census in the 1980s to generate a soil database at a scale of 1:1,000,000 to investigate the spatial distribution of the soil organic carbon stock and density in China. In the southwest of China, they found the highest SOC stock, while the highest average SOC density was located in the northeast of China. Li et al. [16] assessed different land types, soil types, and changes in SOC in the upper, middle, and lower reaches of the Tarim River basin between 2000 and 2020. However, there are relatively few systematic studies on the annual SOC stock, especially at the regional scale [6], which is in part due to the lack of highly accurate, reliable, long-time series SOC datasets. When we use series data to study SOC, we can understand the general evolutionary trends, but we inevitably lose a lot of critical information and ignore internal evolutionary processes, which could lead to the differential overestimation or underestimation of the challenges it faces.

The factors influencing SOC have been broadly studied, with natural and human factors being the two main factors recognized. Natural factors include temperature, precipitation, topography, vegetation cover, and soil type [16,17]. Human factors include land-use change and land management practices (e.g., fertilizer application, mechanization, and crop straw return). Moreover, there is significant spatio-temporal heterogeneity across regions and scales [2,18,19]. A recent study shows that drastic land changes caused by urbanization have gradually increased their impacts on SOC loss [20]. However, there are clear differences in the intensity and direction of land-use change between urban and rural areas, and their impacts on SOC may not be synchronized. To the best of our knowledge, the response of SOC stock to different levels of urbanization has not been fully investigated. Models have been utilized to identify drivers of changes in SOC stock. These models are statistically based, examples of which include quantile regression, the multiple linear regression model, the geographical detector model, and the geographically weighted regression (GWR) model [2,21,22]. However, these models have limitations of their own and usually neglect the spatio-temporal non-stationarity of influencing factors. To overcome the above shortcomings, Huang et al. [23] developed a geographical and temporal weighted regression (GTWR) model that incorporates time- and space-weighted matrixes into regression coefficients, and that performs better in terms of time-varying data prediction than GWR model. The emergence of improved models provides an opportunity to explore the spatial and temporal heterogeneity of the driving force effects of SOC change.

A variety of SOC data products are currently available for different specific years, but most are derived from different methods. There may be large systematic errors when comparing SOC changes between years. Considering the shortcomings of the above studies, we introduce the SOC stock time series dataset covering the period of 2001–2015, and design a new long-term series to analyze how SOC responded to urbanization in Beijing, China, since the onset of the 21st century. Specifically, this study’s main objectives are: (1) to systematically characterize the spatio-temporal changes in land use under an urban-rural gradient (2) to investigate the change trends in SOC stock and density in relation to urbanization; and (3) to identify the main driving forces affecting SOC stock.

## 2. Materials and Methods

### 2.1. Study Area

**Figure 1:**
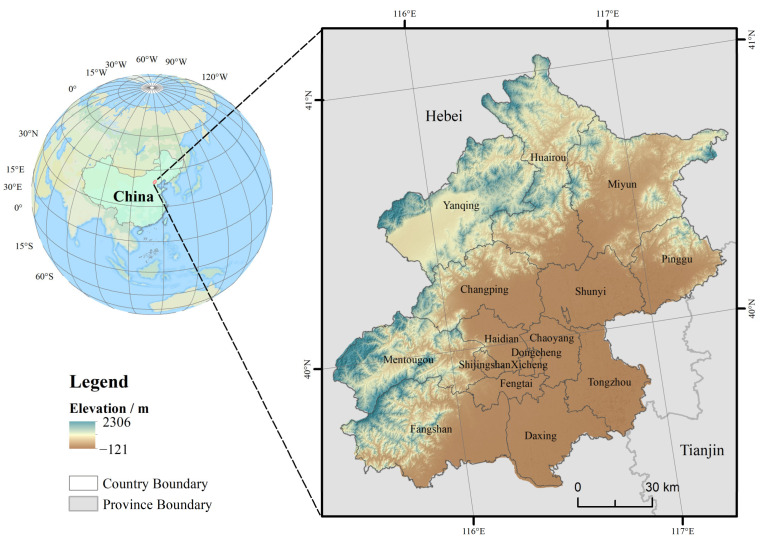
Location of study area.

Beijing, the capital of China, is a rapidly developing super metropolis located in the North China Plain, with high elevation in the northwest and low elevation in the southeast. The topography can be divided into plain areas and mountainous areas (Figure 1). Beijing belongs to the warm temperate zone monsoon climate, with hot and rainy summers as well as cold and dry winters. For example, the annual average values for temperature and precipitation from 1978 to 2020 were 12.94 °C and 544.28 mm, respectively. Beijing has 16 soil types (according to FAO-UNESCO’s 1988), of which Calcic CAMBISOLS, Calcaric FLUVISOLS, Gleyic LUVISOLS, and Calcic LUVISOLS cover more than 70% of the area. The plain areas of Beijing have experienced significant urban sprawl over the past few decades, with a population of 21.89 million in 2020. The urbanization rate of Beijing rose from 77.54% in 2000 to 87.55% in 2020, which is significantly more than the national average in 2020 (63.89%). In order to improve air quality and to protect the ecological environment, the Beijing Municipal Government implemented a large-scale plain afforestation project from 2012 to 2015, with a target of 666 km^2^ of new-planted forest area in the plain [24]. The expansion of impermeable surfaces and man-made greening projects caused by urbanization have reshaped the surface cover and soil environment in Beijing [13,25], manifesting as soil degradation and soil sealing. Therefore, it is representative to explore the changes in the SOC stock under the urban–rural gradient in Beijing.

### 2.2. Data Sources and Processing

#### 2.2.1. Data Sources

This study involves land use data, SOC stock data, digital elevation model (DEM) analogue data, vegetation remote sensing index data, meteorological data, and socio-economic data (Table 1). The Beijing land-use dataset from Xie et al. [26], formed jointly using Continuous Change Detection and Classification algorithm with the Markov random field model, has an overall classification accuracy of 81.93% and contains eight major land-use types. SOC stock data for a 0–30 cm surface were obtained from Wheeler and Hengl [27]. This dataset is currently the most reliable SOC dataset in the world, aggregating a large number of global soil profiles, entering relevant environmental covariates, and modeling using a machine learning framework [6]. The DEM data were derived from the United States Geological Survey (https://www.usgs.gov, accessed on 20 April 2022) and the slope was calculated based on DEM data using the spatial analysis tools in ArcGIS. Soil data were from SoilGrid (https://soilgrids.org/, accessed on 20 April 2022). Population grid data were available from WorldPop (https://www.worldpop.org, accessed on 20 April 2022). Gross domestic product (GDP) grid data were accessed from the Data Center for Resource and Environmental Sciences, Chinese Academy of Sciences (http://www.resdc.cn/, accessed on 20 April 2022). Due to the lack of GDP grid data for 2001, we took data from 2000 instead. Normalized difference vegetation index (NDVI) data, annual mean temperature, and annual mean precipitation data were also directly collected from the Data Center for Resource and Environmental Sciences, Chinese Academy of Sciences (https://www.resdc.cn/, accessed on 20 April 2022). Considering that inconsistency between the data resolution and coordinate system may produce errors in the analysis, we took the resolution of the SOC stock data as a benchmark, used a bilinear interpolation algorithm to resample all data to 250 m, and projected the coordinate system uniformly to WGS 1984 UTM Zone 50 N. The whole process was completed using the ArcGIS software (Version 10.2) [28].

#### 2.2.2. Explanatory Variable Selection

During the process of changing SOC stock, several factors are often involved, including natural and socioeconomic ones. In light of previous studies [2] and data acquisition, explained variable were selected in terms of topography, climate, soil, land use type, vegetation, and socio-economic aspects (Table 2). We generated a 1 km × 1 km grid covering the entire study area and used the zonal statistics tool in ArcGIS software to obtain the mean values for each variable within each grid. Given that land-use type is a discrete variable that cannot be used directly for analysis, we converted it into a continuous variable by counting the area weight of various land-use types within the grid.

### 2.3. Methods

#### 2.3.1. Methodological Flowchart

**Figure 2:**
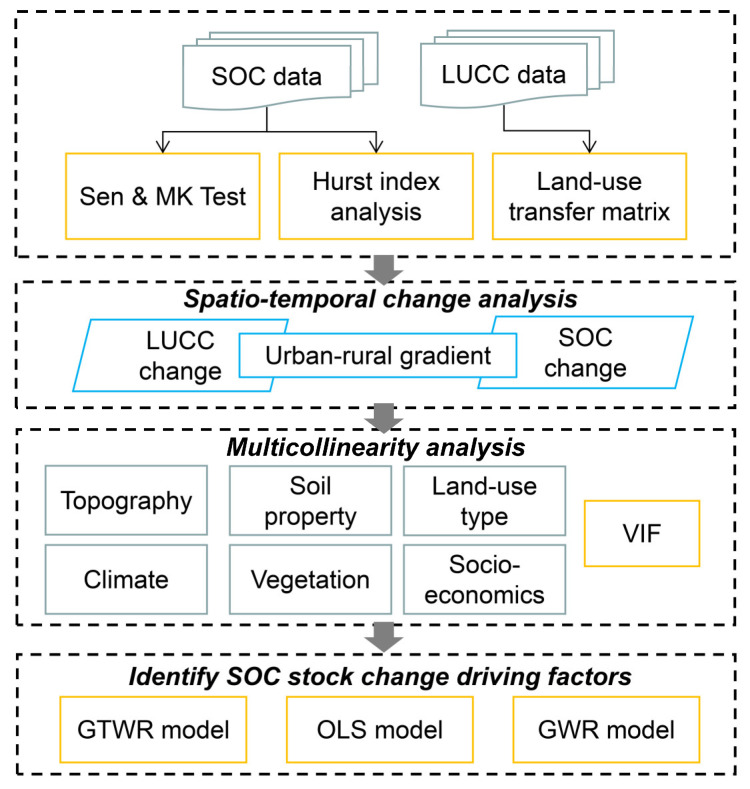
Methodological flowchart.

First, we created concentric buffer zones to construct an urban-rural gradient with Beijing’s urban center. Second, we analyzed the spatial and temporal patterns of land use along the urban-rural gradient using the land-use transfer matrix. Third, we employed the Sen & Mann–Kendall Trend tests to analyze the change characteristics of the SOC under different urban-rural gradients and the Hurst index to predict the future change trends of the SOC. Fourth, multicollinearity analysis was used to eliminate highly correlated variables from topography, climate, soil properties, land-use type, vegetation and socio-economics variables. Finally, the retained variables were modeled using ordinary least squares (OLS) model, GWR model, and GTWR model to obtain the best fitting effect. To improve understanding, a methodological flowchart was shown in Figure 2.

#### 2.3.2. Analysis of Land-Use Changes

Spatially explicit land cover data can clearly reveal the spatio-temporal dynamics of land use, and the land-use transfer matrix is widely used to quantitatively characterize land-use change in specific regions [29,30]. We calculated the changes in land-use type for 2001, 2005, 2010, and 2015 using the Map Algebra tool in ArcGIS software to create a land-use transfer matrix. It is calculated as (1):

(1) $$L_{ij}=\left[\begin{array}{cc} \begin{array}{cc} L_{11} & L_{12}\\ L_{21} & L_{22} \end{array} & \begin{array}{cc} \cdots & L_{1n}\\ \ldots & L_{2n} \end{array}\\ \begin{array}{cc} \vdots & \vdots \\ L_{n1} & L_{n2} \end{array} & \begin{array}{cc} \vdots & \vdots \\ \ldots & L_{nn} \end{array} \end{array}\right]$$

where $L_{ij}$ represents the area of land-use type change from the beginning to the end of the study. n is the number of land-use types.

#### 2.3.3. Analysis of Changes in SOC Stock

##### Sen & Mann–Kendall Trend Test

Trends in the SOC stock from 2001 to 2015 were analyzed using Sen’s trend estimator [31] and the Mann–Kendall test [32,33]. This method is frequently used for the trend analysis of time series data and significance analysis in the fields of hydrology, meteorology, and ecology [31,34,35], and is a non-parametric statistical method. It effectively removes noise from the data. Sen’s trend estimator is calculated as (2):

(2) $$\beta =median\left(\frac{x_{j}-x_{i}}{j-i}\right),\;\forall i<j$$

where β is the change trend of the SOC stock in the time series, in which β>0 indicates that the trend is positive. β<0 indicates that the trend is negative. $x_{i}$ and $x_{j}$ represent the SOC stock in year i and year j, respectively.

The Mann–Kendall test was used to assess the significance of the changes in the SOC stock and is calculated as in (3)–(6):

(3) $$S=\overset{n-1}{\underset{i=1}{\sum }}\overset{n}{\underset{j=i+1}{\sum }}sgn\left(x_{j}-x_{i}\right)$$

(4) $$sgn\left(x_{j}-x_{i}\right)=\{\begin{array}{cc} +1, & if\;x_{j}-x_{i}>0\\ 0, & if\;x_{j}-x_{i}=0\\ -1, & if\;x_{j}-x_{i}<0 \end{array}$$

(5) $$\sigma \left(S\right)=\frac{n\left(n-1\right)\left(2n+5\right)-\sum_{i=1}^{n}t_{i}\left(i\right)\left(i-1\right)\left(2i+5\right)}{18}$$

(6) $$Z_{S}=\{\begin{array}{cc} \frac{S-1}{\sqrt{\sigma \left(S\right)}}, & if\;S>0\\ 0, & if\;S=0\\ \frac{S+1}{\sqrt{\sigma \left(S\right)}}, & if\;S<0 \end{array}$$

where n is the length of the time series dataset, while $x_{i}$ and $x_{j}$ are the values of (j>i) in time series year i  and year j respectively. $sgn\left(x_{j}-x_{i}\right)$ is a sign function. σ(S) is the variance. $t_{i}$ is the number of associations in range i. When $\left|Z_{S}\right|>1.96$, the change trend is considered significant (P<0.05). When $\left|Z_{S}\right|<1.96$, the change trend is considered non-significant (P≥0.05). On the basis of the above results, we classified the trends in the SOC stock into four types: significant improvement (β>0 and P<0.05), slight improvement (β>0 and P≥0.05), slight decrease (β≤0 and P≥0.05) and significant decrease (β≤0 and P<0.05). The calculation of Sen’s trend estimator and the Mann–Kendall test was carried out in R 4.0.2 [36] using the trend package [37].

##### Hurst Index Analysis

An important tool for distinguishing sustainability in time series data is the Hurst index, which was originally proposed by the British scholar Hurst [38]. It can be applied to fields such as economics, meteorology, and hydrology [39]. SOC stock time series data are defined as *t* = 1, 2, ⋯, *n*, and can be calculated as (7)–(11):

(7) $$\bar{f_{\left(\tau \right)}}=\frac{1}{\tau }\sum_{t=1}^{\tau }f_{\left(t\right)},\tau =1,2,\cdots ,n$$

(8) $$X_{\left(t,\tau \right)}=\sum_{t=1}^{t}\left(f_{\left(t\right)}-\bar{f_{\left(\tau \right)}}\right),1\leq t\leq \tau$$

(9) $$R_{\left(\tau \right)}=\underset{1\leq t\leq \tau }{\max}X_{\left(t,\tau \right)}-\underset{1\leq t\leq \tau }{\min}X_{\left(t,\tau \right)},\;\tau =1,\;2,\cdots ,n$$

(10) $$S_{\left(\tau \right)}={\left[\frac{1}{\tau }\sum_{t=1}^{\tau }{\left(f_{\left(t\right)}-\bar{f_{\left(\tau \right)}}\right)}^{2}\right]}^{\frac{1}{2}},\tau =1,\;2,\cdots ,n$$

(11) $$\frac{R_{\left(\tau \right)}}{S_{\left(\tau \right)}}={\left(c\tau \right)}^{H}$$

The value of H is calculated by fitting the equation $\log{\left(R/S\right)}_{n}=a+H\times \log\left(n\right)$ using the least squares method, where H represent the Hurst index and takes values in the range of ϵ (0, 1). When  H=0.5, it indicates that the time series of the SOC stock is random and unsustainable. When  1>H≥0.5, it indicates positive sustainability, and the future trend in the SOC stock will be consistent with the present. When  0<H<0.5, it indicates negative sustainability, and the future trend in the SOC stock will be opposite to the current one. $\bar{f_{\left(\tau \right)}}$ is the average of the time series. $X_{\left(t,\tau \right)}$ is the cumulative deviation at time *t*. $R_{\left(\tau \right)}$ is the range of the time series. $S_{\left(\tau \right)}$ is the standard deviation of the time series. The calculation of the Hurst index was carried out in R 4.0.2 using the reservoir package [40].

#### 2.3.4. Multicollinearity

Multicollinearity in a regression model implies that several specific explanatory variables are highly linearly correlated with each other, which may lead to bias in explaining the effects of other explanatory variables. To eliminate this phenomenon, we used the variance inflation factor (VIF), which is an indicator of the severity of multicollinearity. It is assumed that variables with VIF values greater than 10 are multicollinearity variables and should be removed from the model [41].

#### 2.3.5. Regression Models

##### OLS and GWR Models

OLS model is the classical method used to analyze the linear relationship between multiple explanatory variables and the explained variable, estimating the global parameters using the least squares method. Its formula is as follows [42]:

(12) $$y=\beta_{0}+\sum_{k=1}^{\rho }\beta_{k}x_{k}+\varepsilon \;\;k=1,2\cdots \rho$$

where y is the explained variable value; $\beta_{0}$ is a constant; $\beta_{k}$ is the regression coefficient of the explanatory variable; ρ is the number of the explanatory variable; and ε is the error item.

However, OLS model do not take the spatial non-stationarity of the influencing variables into account adequately. GWR model improves on the shortcomings of traditional linear regression models by incorporating location information into the regression parameters. The formula is as follows [41]:

(13) $$y_{s}=\beta_{0}\left(q_{s},u_{s}\right)+\sum_{k=1}^{\rho }\beta_{k}\left(q_{s},u_{s}\right)x_{sk}+\varepsilon_{s}\;\;k=1,2\cdots \rho$$

where for each region *s*, $y_{s}$ is the explained variable value of region s; $\left(q_{s},u_{s}\right)$ is the spatial coordinate of region *s*; $\beta_{0}\left(q_{s},u_{s}\right)$ is the intercept at region *s*; $x_{sk}$ is the observed value of region s; $\beta_{k}\left(q_{s},u_{s}\right)$ is the local estimated coefficient of the explanatory variable $x_{sk}$; ρ is the number of explanatory variable; and $\varepsilon_{s}$ is the error item.

###### GTWR Model

SOC stock changes are related to environmental variables using a variety of regression models, such as linear regression models and GWR model [21,43]. As a temporal extension of GWR model, GTWR model can capture the spatio-temporal non-stationarity of the variables, reflecting the spatio-temporal heterogeneity of them and bringing the regression results closer to the reality of the situation [23]. The model is calculated as (14):

(14) $$y_{s}=\beta_{0}\left(q_{s},u_{s},t_{s}\right)+\sum_{k=1}^{\rho }\beta_{k}\left(q_{s},u_{s},t_{s}\right)x_{sk}+\varepsilon_{s}\;\;k=1,2\cdots \rho$$

where for each region *s*; $y_{s}$ is the explained variable value of region *s*; $x_{sk}$ is the kth explanatory variable of region *s*; $\beta_{0}$ is the intercept of the coordinate points $\left(q_{s},u_{s},t_{s}\right)$; $q_{s}$, $u_{s}$, and $t_{s}$, are the longitude, latitude, and time of region *s*, respectively; $\beta_{k}$ is the local estimated coefficient of the coordinate points $\left(q_{s},u_{s},t_{s}\right)$; ρ is the number of explanatory variable; and $\varepsilon_{s}$ is the error item. The estimated parameters can be expressed as follows:

(15) $$\hat{\beta }\left(q_{s},u_{s},t_{s}\right)={\left[X^{T}W{\left(q_{s},u_{s},t_{s}\right)}^{2}X\right]}^{-1}X^{T}W\left(q_{s},u_{s},t_{s}\right)Y$$

where the spatio-temporal weight matrix $W\left(q_{s},u_{s},t_{s}\right)=diag\left(w_{s1},w_{s2},\cdots ,w_{sn}\right)$ and *n* is the number of observations. $w_{sk}\left(1\leq k\leq n\right)$ is the space-time distance decay functions of $\left(q_{s},u_{s},t_{s}\right)$, which is calculated as follows (16)–(17):

(16) $$w_{sk}=\exp\left[-\frac{{\left(d_{sk}^{ST}\right)}^{2}}{h^{2}}\right]$$

(17) $$d_{sk}^{ST}=\sqrt{\lambda \left[{\left(u_{s}-u_{k}\right)}^{2}+{\left(q_{s}-q_{k}\right)}^{2}\right]+\sigma {\left(t_{s}-t_{k}\right)}^{2}}$$

where $w_{sk}$ is the spatio-temporal weight matrix; $d_{sk}^{ST}$ is the spatio-temporal distance between the observation point s and the observation point *k*; and h is the bandwidth, the value of which affects the goodness-of-fit of the model. In order to obtain an appropriate bandwidth, adaptive bandwidth is used in this study and the selection criterion refers to the Akaike information criterion (AIC).

#### 2.3.6. Urban–Rural Gradient Construction

**Figure 3:**
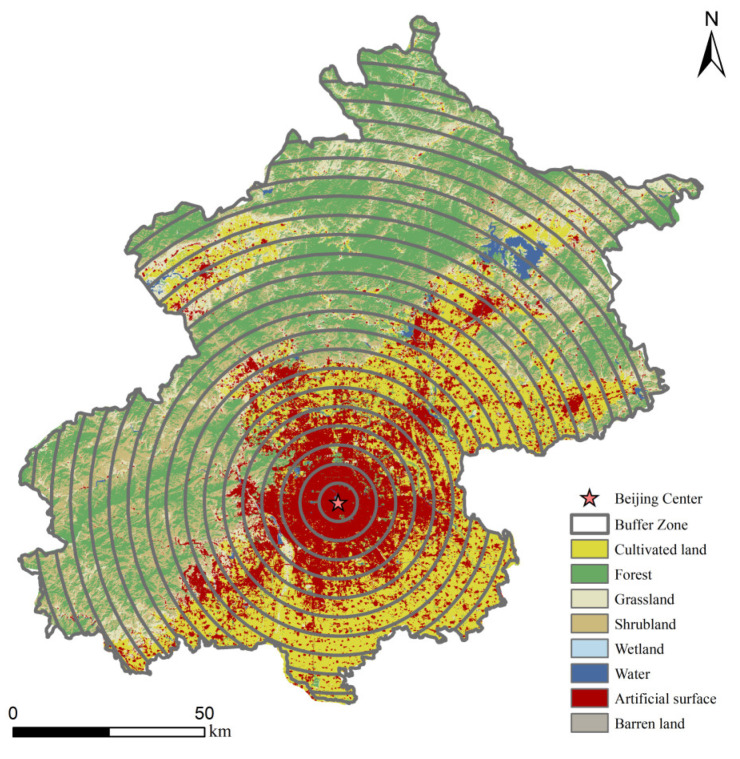
Urban-rural gradient in Beijing.

The urban–rural gradient, as a replacement for spatio-temporal methods [44], plays an important role in urbanization-related studies lacking time-series data. For instance, some researchers investigate the effects of urbanization on soils, biodiversity, ecosystem services, vegetation phenology, and the fragmentation of cultivated land [24,45,46,47,48]. In recent years, several scholars have also applied the urban-rural gradient to studies on topics related to spatio-temporal variation. In this study, 26 concentric buffer zones were established in a 5 km width, with Tiananmen Square in Beijing as the center of the circle, (Figure 3) to analyze the dynamics of land use and SOC stock.

## 3. Results

### 3.1. Spatio-Temporal Characteristics of Land-Use Change

**Figure 4:**
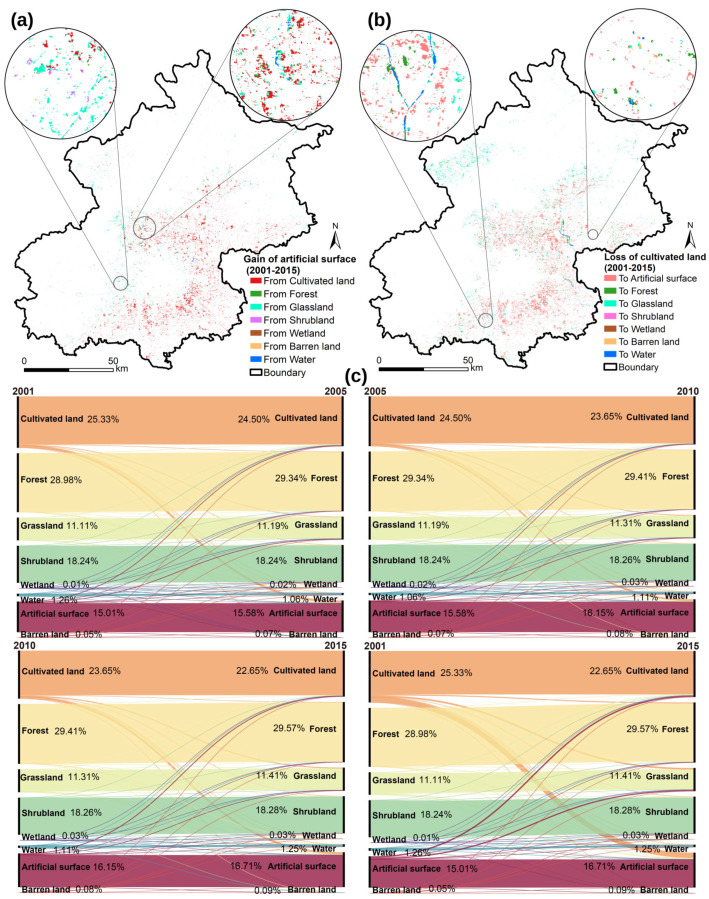
Spatio-temporal changes of land-use in Beijing from 2001 to 2015. The sources of newly gained artificial land surfaces (**a**) and the destination of lost cultivated land (**b**) between 2001 and 2015. The Sankey diagram (**c**) shows the land-use changes between the previous period (**left**) and the next period (**right**). The width of the curve reflects the magnitude of land-use type change. The ratios represent the proportion of land-use types to the total land area in the region at different periods.

Forest, cultivated land, shrubland, and artificial surface are the main land use types in Beijing (Table 3), and there are marked differences in the changes in various land types (Figure 4). The area of cultivated land decreased significantly during the study period, from 4156.89 km^2^ in 2001 to 3717.62 km^2^ in 2015, and the reduction has gradually accelerated. The loss of cultivated land is mainly located in the peri-urban areas of the plain (Figure 4a), with most turning into artificial surfaces (64.57%), grasslands (19.69%), and forests (12.33%). The area of forest, grassland and artificial surfaces increased significantly between 2001 and 2015, adding 97.12 km^2^, 49.25 km^2^, and 278.91 km^2^, respectively. Among these, the new artificial surface is mainly from cultivated land (79.20%) and grassland (14.08%) and is widely distributed in the plains as well as in the mountainous areas of the northwest (Figure 4b). It is worth noting that although the new area of wetland and barren land is relatively small, the growth rate is much higher than that of other land types, namely 137%, and 78%.

**Figure 5:**
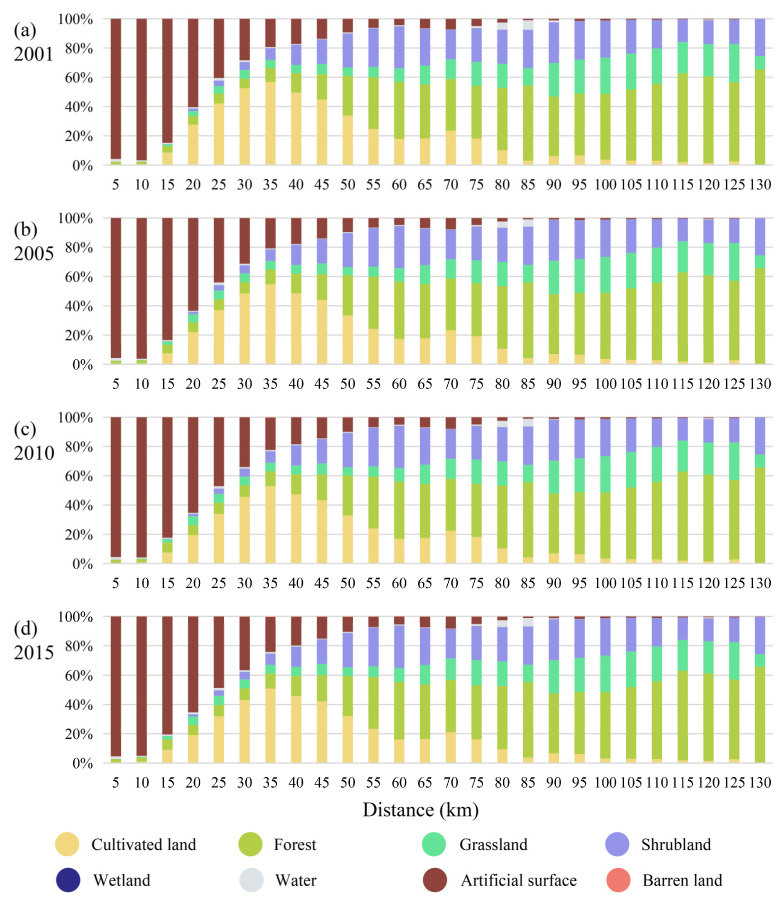
Changes in land-use structure along the urban-rural gradient from 2001 to 2015: (**a**) 2001; (**b**) 2005; (**c**) 2010; (**d**) 2015.

A buffer zone was established with a width of 5 km outwards as a reference for the gradient of urbanization, with 0 to 130 km representing a gradual decrease in the level of urbanization. Figure 5 shows the response of the land-use composition of Beijing to the level of urbanization in different periods. In general, the proportion of artificial surface decreases as the level of urbanization decreases, with the proportion of artificial surface below 50% beyond 25 km. The proportion of cultivated land rises and then falls, with the proportion of it starting to fall beyond 35 km. The trend for forest and grassland is the opposite of that for artificial surface, with the proportion of both increasing as the level of urbanization decreases. The proportion of grassland decreases significantly at 130 km. The trend in shrubland is relatively complex, first experiencing a distinct expansion and then remaining dynamically stable. The gradient effect of changes in other land types is not significant.

### 3.2. Spatio-Temporal Variation Characteristics of SOC Stock

**Figure 6:**
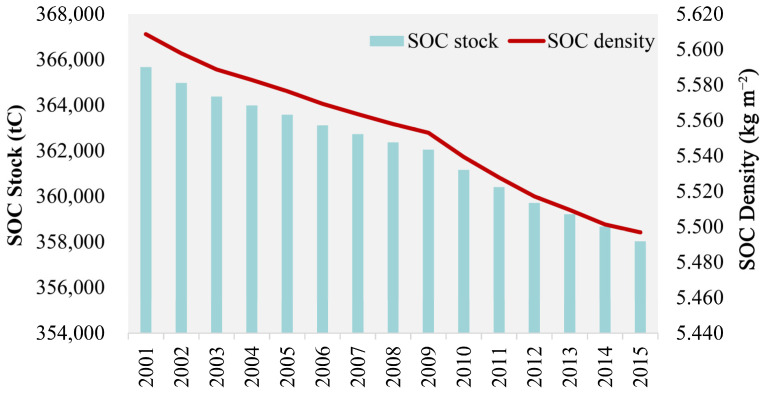
Temporal changes in SOC stock and SOC density at 0–30 cm in Beijing from 2001 to 2015.

**Figure 7:**
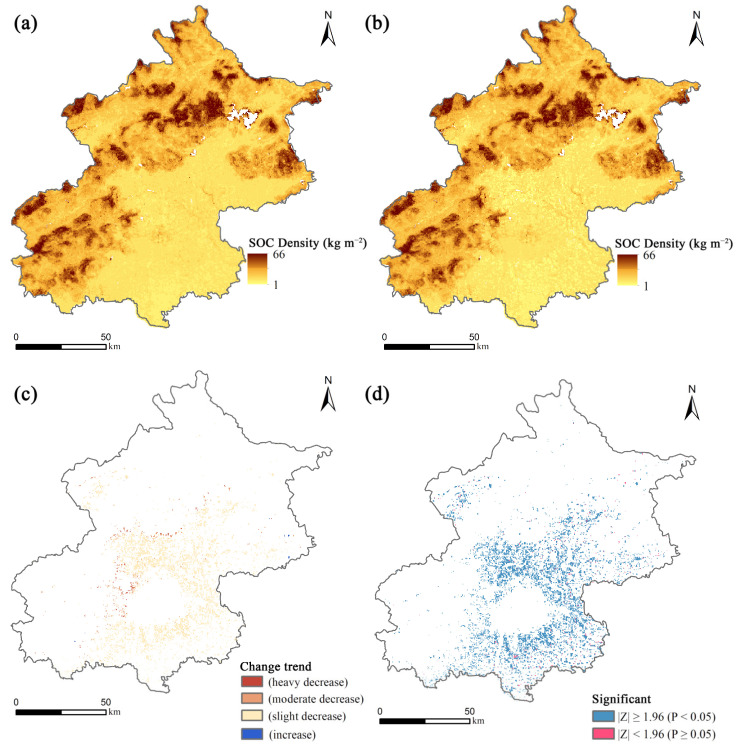
Spatial changes in SOC density at 0–30 cm in Beijing from 2001 to 2015: (**a**) 2001; (**b**) 2015; (**c**) the changing trend of SOC density; (**d**) the results of the significance test.

**Figure 8:**
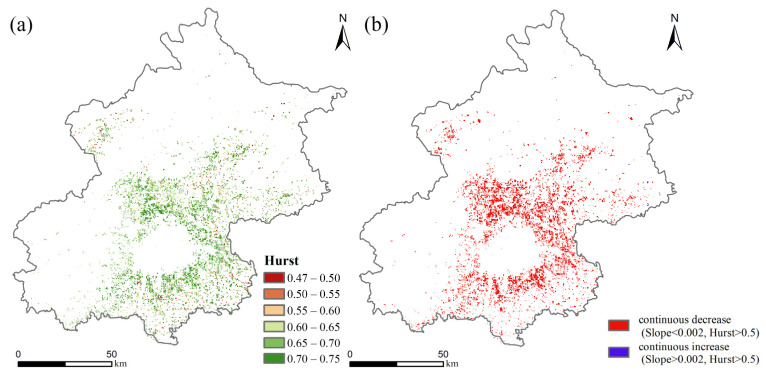
Future trends of SOC density at 0–30 cm in Beijing area: (**a**) Hurst index; (**b**) type of future trends of SOC density.

From 2001 to 2015, Beijing experienced a continuous decrease in both SOC stock and SOC density in the surface 0–30 cm (Figure 6), with a decrease of 7651.50 t C in the total SOC stock and of 0.112 kg m^−2^ in SOC density. The year 2009 was a key turning point, with a higher rate of SOC stock and density loss from 2010 to 2015 than from 2001 to 2009. The distribution of the SOC density shows striking spatial heterogeneity (Figure 7), with some high values mainly in the northern and western mountainous areas of Beijing and lower values in the plains as a whole. There was a significant decrease in the SOC density in the plains during the study period, with most experiencing moderate decreases of −0.143 to −0.002 kg m^−2^ and heavy decreases of −0.500 to −0.224 kg m^−2^. In order to investigate the future trends in SOC density, we used the Hurst index to implement pixel by pixel (250 m × 250 m) calculations and to obtain the spatial visualization results of the Hurst index (Figure 8). The average Hurst index of the SOC density in Beijing is 0.665, and the areas with high Hurst index values (>0.5) are mainly located around the central city, indicating that the current trend of SOC density changes in these areas will be continuous. By overlaying the results of annual trends, we found that the SOC density will decline further in most plains of Beijing in the future, with only scattered areas in the northeast rising (Figure 8b).

**Figure 9:**
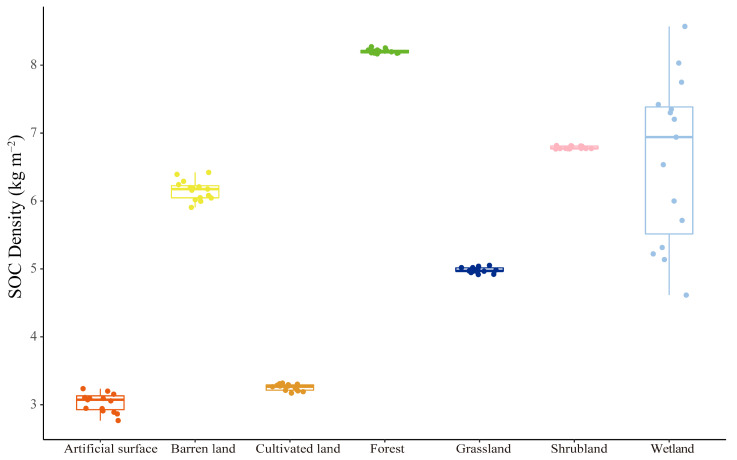
The SOC density at 0–30 cm in diverse land-use types in Beijing from 2001 to 2015. Water was excluded since its SOC density was considered to be 0. The different colors represent the average SOC density of different land use types.

**Figure 10:**
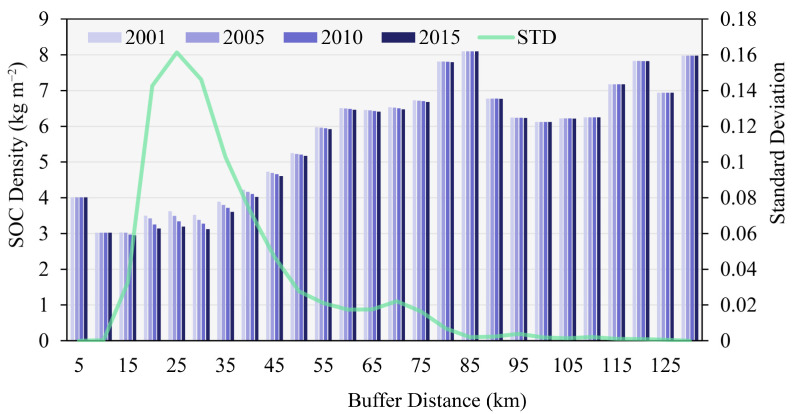
Changes in the SOC density at 0–30 cm along an urban–rural gradient from 2001 to 2015. STD is the standard deviation of SOC density.

The SOC density of various land-use types in Beijing have remained stable over the past 15 years (Figure 9). Only wetlands showed large annual fluctuations in SOC density. Among different land types, the means of the SOC density are ranked as follows: forest > wetland > shrubland > barren land > grassland > cultivated land > artificial surface. Figure 10 shows the dynamics of the SOC density under various urban–rural gradients. In general, the SOC density decreases and then increases as the level of urbanization decreases, with two peak areas at 80–85 km and 130 km, where the SOC density is much higher than it is in other areas. Significantly, the most dramatic annual changes in SOC density took place at 20–40 km, where there was a remarkable decrease.

### 3.3. Driving Factors Analysis of SOC Stock

#### 3.3.1. Comparison of Model Performance

We eliminated variables with a VIF greater than 10 by checking for multicollinearity, and we used Distance, Slope, Per, POP, Forest, Grassland, and SILT to explain SOC stock changes. Table 4 summarizes the results of fitting OLS, GWR, and GTWR models. We found that GTWR model had the lowest AICc and the highest R^2^ (0.951), indicating that the fit of this model was significantly better than that of OLS model (0.635) and slightly better than that of GWR model (0.931). Hence, when analyzing the driver of changes in SOC stock, GTWR model would be more appropriate.

#### 3.3.2. Analysis of Drivers Using GTWR Model

**Figure 11:**
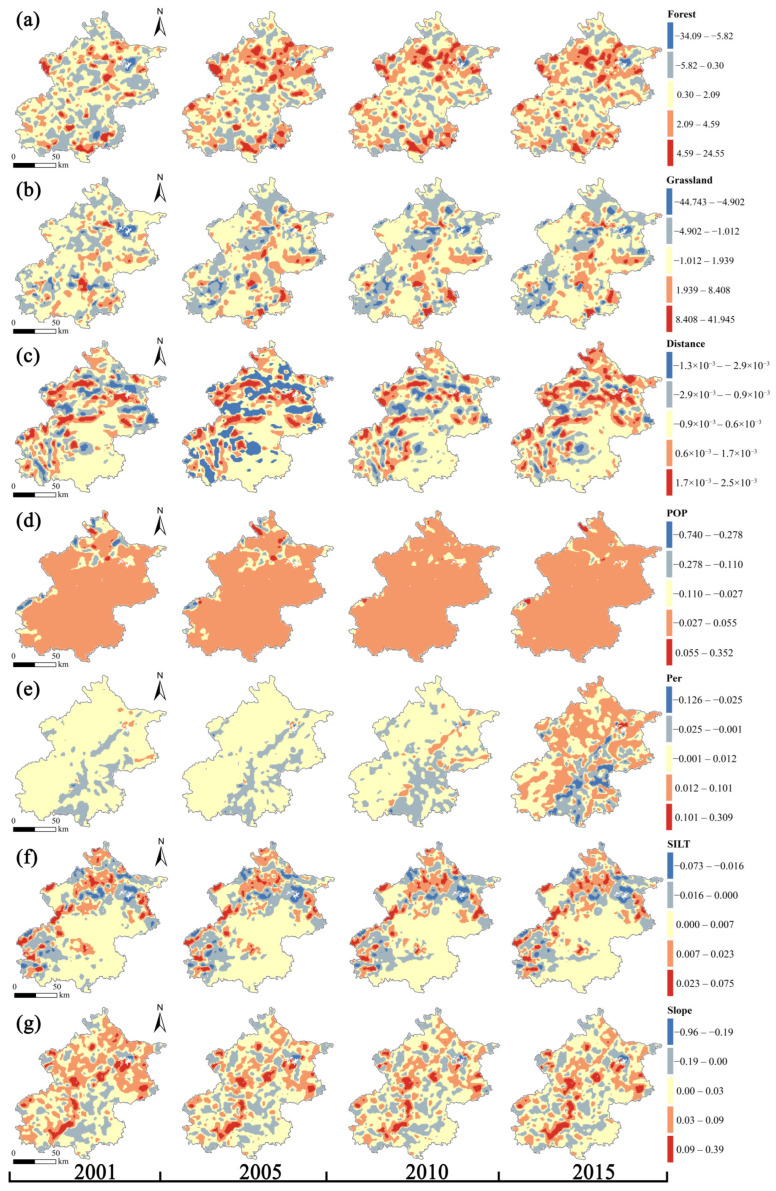
Local regression coefficients of explanatory variables for changes in SOC stock from 2001 to 2015: (**a**) Forest; (**b**) Grassland; (**c**) Distance; (**d**) POP; (**e**) Per; (**f**) SILT; and (**g**) Slope.

**Figure 12:**
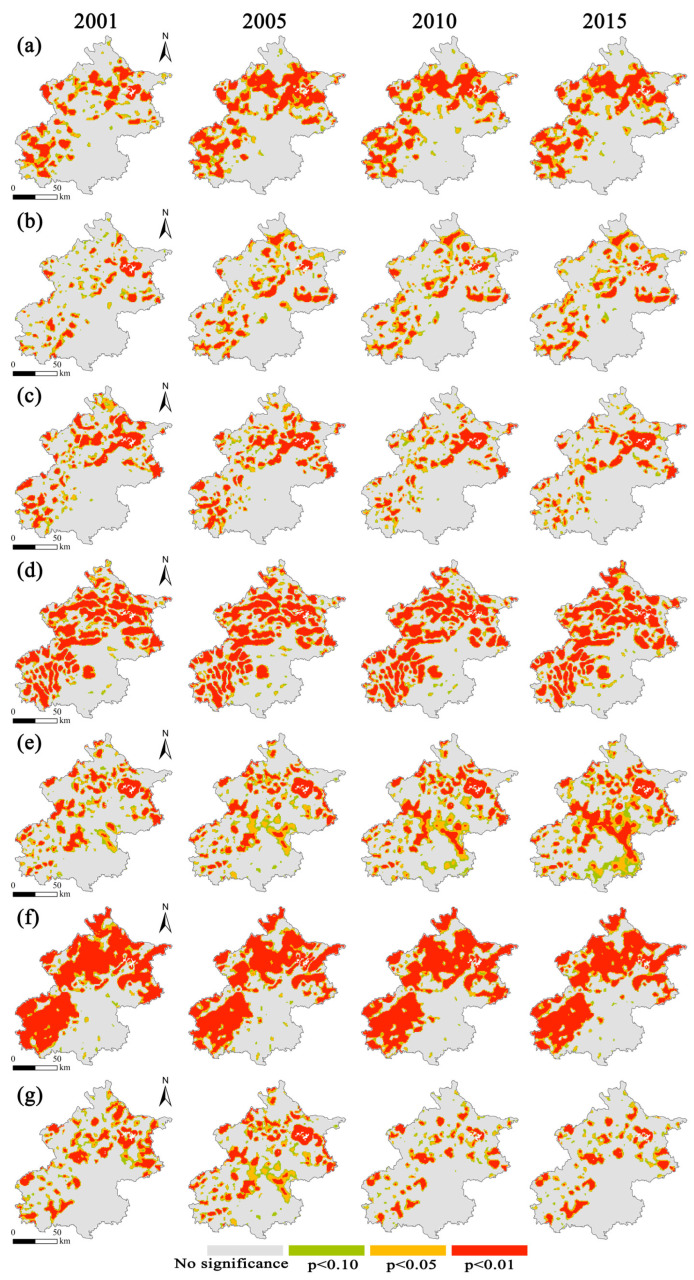
Significance of regression coefficients for explanatory variables: (**a**) Forest; (**b**) Grassland; (**c**) POP; (**d**) Distance; (**e**) SILT; (**f**) Per; and (**g**) Slope.

We investigated the spatio-temporal variability of the factors affecting SOC stock from 2001 to 2015 using the GTWR model. Figure 11 shows how the local regression coefficients of the explanatory factors are estimated spatially by GTWR model, and the significance test results show that there are spatio-temporal disparities in the significant effects of the explanatory variables on SOC stock (Figure 12). The positive effects of Forest, Distance, and SILT on SOC stock were primarily distributed in the mountainous areas in northern and western Beijing, while the positive effects of Grassland were mainly located in the central and north of Beijing. Slope had different positive and negative effects on SOC stock, with the former being more prevalent in Beijing’s western area. On the contrary, the positive effects of POP and Per on SOC stock were mainly located in Beijing’s northern area. The percentage of grids with positive regression coefficients for each explanatory variable is shown in Table 5, which illustrates the general trend of the explanatory variables’ positive impact on SOC stock. It is obvious that over time, the positive effects of Forest, Grassland, Distance, and POP on SOC stock increase dramatically, whilst the positive effects of Per and SILT on SOC stock stay essentially consistent. Slope, on the other hand, has a diminishing positive effect over time.

## 4. Discussion and Impact

Globally, urbanization is on the rise in both developed and developing countries, and the impact on the environment is expected to increase. Recent studies indicate that the global urban land area increased from 450.97 million km^2^ in 1990 to 747.05 million km^2^ in 2010 [49], and the top 3 countries contributing the most to urban land growth worldwide are China, the United States, and India. There is now ample evidence that urbanization can have significant effects on many of the physical and chemical properties of soils [50], including pH, bulk density, moisture, organic matter, heavy metal pollution, etc. Some small-scale studies have investigated changes in the SOC in urban cores, urban-rural transition zones and rural areas [11,13,51]. For instance, Vasenev et al. [52] discovered that urbanization had a positive net effect on the SOC stock in the Moscow region for 2014–2048 due to increased SOC in low-fertility topsoil and subsoil. Yan et al. [20] investigated changes in the urban SOC stock in Urumqi, China from 1990 to 2010 and found that the artificial surface expansion of the city resulted in SOC stock loss of 0.77 Tg. Most studies do not take into account the differences in urbanization levels across regions and do not track the dynamics of SOC over time in the same region. As a result, the conclusions of many studies tend to be mixed, which is not conducive to our understanding of the dynamics of SOC.

Taking advantage of the land-use transfer matrix, time series trend analysis, and urban–rural gradients, our study provides a different approach to quantify the impact of urbanization on land and SOC stock and SOC density as well as deepens our understanding of it. Our study shows that the total SOC stock and average SOC density continue to decline in highly urbanized metropolitan areas, and the rate of decline gradually accelerating. SOC changes within metropolitan region are uneven, usually declining significantly in plains while remaining stable in mountains. It is noteworthy that SOC density is most sensitive to urbanization in suburban areas, rather than in urban cores or remote rural areas. According to (Figure 9), the stability of SOC density in suburban areas is poor and the risk of loss is high. This finding is generally consistent with comparable case studies [53,54] indicating that urbanization has a variable impact on SOC stock, with rapid urbanization leading to significant losses of SOC in suburban areas. Urbanization has led to the spreading of built-up areas and transport facilities to suburban areas and the conversion of a large amount of cultivated land to urban use, resulting in soil compaction and sealing as well as increased organic carbon loss [11]. Due to the difference in land rents, suburban areas around metropolitan areas tend to develop more profitable vegetable and fruit farming in place of traditional field crops. As a result of these land-use practices, soil properties and water balance are altered, affecting soil organic matter accumulation and decomposition and, leading to SOC loss [55]. Moreover, the soil in the urban core is sealed and rarely cultivated. The landscape types in rural areas are mainly forests and grasslands, which are less affected by human activities. Climate change is a key factor driving SOC dynamics. Temperature is inversely proportional to soil organic matter content according to several studies [56], since lower temperatures inhibit soil microbial respiration and reduce organic matter decomposition. Precipitation is positively related to SOC content because rainfall promotes vegetation growth and vegetation-derived carbon increases soil organic matter input [57]. Additionally, climate change will indirectly affect SOC content through changes in land use. As global warming improves thermal conditions at high latitudes, forests, grasslands, and wetlands with high SOC contents are being reclaimed for crops, and their SOC content is decreasing [57,58]. In practice, SOC’s response to climate change varies from region to region [56]. The actual effect of climate change on SOC depends on many factors, including climatic conditions, soil management practices, and soil properties. SOC is closely related to the achievement of the SDGs. Losses in SOC not only reduce land productivity and crop yield [59], which hinder the achievement of the zero hunger target, but also causes more natural land to be cleared for crop production, which exacerbates the degradation of terrestrial ecosystems. SOC losses release large amounts of greenhouse gases, which may offset anthropogenic mitigation efforts [60], which may worsen the effects of global climate change on SOC. In this study, precipitation in Beijing did not change significantly, so the SOC in the urban core and rural areas remained stable. In addition, some studies report that with urbanization moving forward, the level of surface SOC stock in the urban core decreases and then increases, shifting from a carbon source to a carbon sink [54]. However, our study shows that the SOC stock in the urban core remained largely stable, which is not consistent with some previous studies. The difference in results may be due to the different sources of SOC data used in the study. Generally, the SOC stock is higher in internal urban green spaces (e.g., parks, green belts, and home gardens) [51] than on artificial surfaces. As the level of urbanization increases, the area of green space in the internal urban also expands, leading to an increase in SOC stock in the urban core. Therefore, SOC data collected by sampling in different landscapes within the urban core can reflect a trend of decreasing and then increasing the SOC stock in the urban core. In this study [6], SOC data were estimated using a model that ignored internal urban landscape changes and treated urban areas as homogeneous artificial surfaces.

Some previous studies have used the structural equation model, multiple linear regression model, and GWR model to determine the relationship between SOC stock and the drivers of change [2,21,22,55], but they did not consider the spatio-temporal non-stationarity of SOC stock and drivers. This study used GTWR model and determined the spatio-temporal heterogeneity of the drivers influencing SOC stock at the grid scale for four periods: 2001, 2005, 2010, and 2015. Our study found that climate, land-use type, topography, soil properties, and socio-economics are important factors controlling SOC stock. Climatic factors mainly refer to precipitation; topographic factors mainly refer to slope; land-use types mainly refer to forest and grassland; soil properties mainly refer to SILT; and socio-economic factors mainly refer to population and distance. The influence of the various drivers on SOC stock varies across time and space. The positive effects of Forest, Grassland, POP, and Distance on SOC stock increase gradually, while the positive effects of Per, Slope and SILT are smaller or even decreasing slightly. This is consistent with previous studies [11] showing that human interventions, especially urbanization, have a stronger impact on SOC stock than natural interventions. The Beijing government should develop a differentiated and flexible strategy to manage SOC. The positive effects of forests, grasslands, the density of population, and the distance from urban centers on SOC stock were shown to grow over time in our study. Hence, the mountainous regions of northern and western Beijing should increase forest and grassland cover; strictly prevent human activities from degrading natural forests, wetlands and shrubs, and increase regional SOC accumulation in order to offset the loss of SOC in the plains. The North China Plain, where Beijing is located, has limited water resources, and large-scale afforestation of the plain will exacerbate the current water shortage, although afforestation has the potential to increase SOC accumulation. A strict control of urban sprawl in plain areas is therefore necessary, and infrastructure construction should avoid or reduce land occupation in areas with high SOC density. Furthermore, cultivated land in suburban areas of metropolitan cities should moderately increase fertilization and returning crop residue to the field to increase SOC accumulation, encourage crop rotation from monoculture, and enhance dynamic monitoring of SOC.

Considering the data and model used in this study, we acknowledge two limitations. Firstly, the factors influencing the determination of SOC stock have become more complex with the increased impact of human activities on soils. As high-resolution socio-economic grid data are difficult to obtain, in this study only population and distance from the city were selected as socio-economic explanatory variables; more socio-economic factors will need to be considered in future studies. Secondly, the use of the Sen & Mann–Kendall trend tests has some restrictive prerequisites, such as the independent nature of the data series, the normality of the distribution, and the length of the data [61]. To compensate for the limitations presented by the Sen & Mann–Kendall, other emerging trend analysis methods such as the innovative trend analysis method (ITA) and multidimensional ensemble empirical model decomposition (MEEMD) [62] can be considered for future analysis of SOC dynamic changes.

## 5. Conclusions

In the first 15 years of the 21st century, Beijing’s land use pattern underwent dramatic changes due to urbanization. Among its most notable features are the expansion of artificial surfaces and the loss of cultivated land. Most cultivated land has been replaced with artificial surfaces, which are mainly in the plains. At the same time, the SOC stock and density show a declining trend, with a cumulative loss of 7651.50 t C and an average annual decline of 0.112 kg m^−2^ over 15 years, spatially located mainly around the central city. Both land use structure and SOC density respond differently to urbanization. The response trend varies among land types. Taking artificial surfaces, for example, the proportion of it decreases as the level of urbanization decreases. As the level of urbanization decreases, the average SOC density experiences a decline followed by an increase, with the most pronounced decline occurring at 20–40 km from the urban center. Climate, topography, land use type, soil properties, and socio-economics are the main drivers influencing SOC stock, with significant spatio-temporal variation in the relationship between different drivers and SOC stock. In the future, we suggest that policymakers pay high attention to changes in SOC in suburban areas and strengthen the monitoring of SOC stock dynamics. The spatio-temporal heterogeneity of influencing factors should be incorporated into regional SOC management, which can significantly improve the effectiveness of related policies and mitigate SOC losses.

## Figures and Tables

**Table 1:** Data information [26,27].

Dataset	Year	Spatial Solution	Source
Land use data	2001–2015	30 × 30 m	Xie et al. (2022)
SOC stock data	2001–2015	250 × 250 m	Wheeler and Hengl (2018)
DEM	2009	30 × 30 m	The United States Geological Survey
Soil data	2017	250 × 250 m	SoilGrid
Population	2001–2015	1 × 1 km	WorldPop
GDP	2000–2015	1 × 1 km	Chinese Academy of Sciences
NDVI	2001–2015	1 × 1 km	Chinese Academy of Sciences
Temperature	2001–2015	1 × 1 km	Chinese Academy of Sciences
Precipitation	2001–2015	1 × 1 km	Chinese Academy of Sciences

**Table 2:** Potential explanatory variables affecting SOC stock.

Variables	Indicators	Describe	Is It Dynamic
Topography	Elevation	Average elevation, m	No
	Slope	Average slope, degree	No
Climate	Tem	Average annual temperature, °C	Yes
	Per	Average annual precipitation, mm	Yes
Soil property	CEC	Cation exchangeable capacity, cmol/kg	No
	SILT	Silt content, %	No
	CLAY	Clay content, %	No
	pH	Soil pH	No
Land-use type	Cultivated land	Cultivated land rate, %	Yes
	Grassland	Grassland rate, %	Yes
	Artificial surface	Artificial surface rate, %	Yes
	Forest	Forest rate, %	Yes
	Shrubland	Shrubland rate, %	Yes
	Wetland	Wetland rate, %	Yes
	Barren land	Barren land rate, %	Yes
	Water	Water rate, %	Yes
Vegetation	NDVI	Annual Normalized Difference Vegetation Index	Yes
Socio-economics	Distance	Euclidean Distance to the urban center, km	No
	POP	Annual population density, person km^−2^	Yes
	GDP	Annual Gross Domestic Product, yuan km^−2^	Yes

**Table 3:** Statistical results of land-use change in Beijing for 2001 to 2015 (unit: km^2^).

Year	Cultivated Land	Forest	Grassland	Shrubland	Wetland	Water	Artificial Surface	Barren Land
2001	4156.89	4756.36	1822.83	2993.84	2.38	206.90	2464.11	8.60
2005	4021.72	4815.89	1836.58	2993.47	2.92	173.41	2556.30	11.61
2010	3881.10	4826.72	1855.40	2997.46	5.02	182.09	2650.25	13.88
2015	3717.62	4853.48	1872.08	2999.60	5.66	205.10	2743.02	15.35
2001–2005	−135.17	59.53	13.75	−0.37	0.54	−33.49	92.19	3.01
2005–2010	−140.62	10.83	18.82	3.99	2.10	8.69	93.95	2.27
2010–2015	−163.48	26.76	16.68	2.14	0.64	23.00	92.77	1.48
2001–2015	−439.27	97.12	49.25	5.76	3.27	−1.80	278.91	6.76

**Table 4:** Performance results for different models.

Model	R^2^	Adjusted R^2^	AICc	Bandwidth	Residual Sum of Squares
OLS	0.635	0.635	253,079.66	-	-
GWR	0.948	0.931	156,289.41	17	28,686.46
GTWR	0.963	0.951	135,475.98	17	20,871.31

**Table 5:** Proportion of grids with positive coefficients on GTWR model variables.

Variables	2001	2005	2010	2015
Forest	76.94%	85.47%	86.23%	87.29%
Grassland	45.84%	48.55%	49.00%	51.46%
Per	78.58%	75.73%	82.20%	78.31%
Slope	75.43%	68.86%	67.65%	70.31%
POP	36.19%	36.80%	39.32%	40.46%
Distance	44.30%	46.61%	43.77%	55.51%
SILT	73.27%	71.87%	72.36%	74.19%

## Data Availability

The data presented in this study are available upon reasonable request from the corresponding author.
